# Long-term follow-up of secondary amyloidosis patients treated with tumor necrosis factor inhibitor therapy: A STROBE-compliant observational study

**DOI:** 10.1097/MD.0000000000007859

**Published:** 2017-08-25

**Authors:** Sinem Nihal Esatoglu, Gulen Hatemi, Serdal Ugurlu, Aycan Gokturk, Koray Tascilar, Huri Ozdogan

**Affiliations:** a Division of Rheumatology, Department of Internal Medicine, Cerrahpasa Medical School, Istanbul University; b Department of Internal Medicine, Cerrahpasa Medical School, Istanbul University, Istanbul, Turkey.

**Keywords:** amyloidosis, ankylosing spondylitis, anti-TNFs, Behçet disease, FMF, rheumatoid arthritis

## Abstract

There are no treatment modalities, which were proven to prevent the deposition of amyloid, proteinuria, and loss of renal function due to amyloidosis. Anti-tumor necrosis factor agents (anti-TNFs) were shown to decrease the production of serum amyloid A protein.

We aimed to evaluate the long-term efficacy and safety of anti-TNFs in secondary (AA) amyloidosis patients treated in a single center.

Thirty-seven patients with AA amyloidosis were started an anti-TNF for AA amyloidosis between March 2001 and June 2008 and followed until May 2016 unless deceased. They were surveyed for the endpoints of death, development of end-stage renal disease (ESRD), switch to another agent due to worsening of amyloidosis and adverse events.

Among the 37 patients, 12 (32%) had died, 9 (24%) had ESRD, and 8 (22%) had started another group of biologic due to worsening of amyloidosis indicated by an increase in proteinuria, 5 (14%) patients are still doing well with anti-TNFs, and 3 (8%) are off treatment at the end of a median follow-up of 10 (interquartile range [IQR]: 5.5–10.5) years since the start of anti-TNFs and 10 (IQR: 8–13) years since the diagnosis of AA amyloidosis. Most common serious adverse events were sepsis and thrombotic events observed in 8 and 4 patients, respectively.

Treatment with anti-TNFs may be associated with a higher survival rate compared with historic cohorts of AA amyloidosis, especially when started early with a lower serum creatinine level at baseline. Caution is needed regarding serious adverse events, especially infections.

## 1 Introduction

The amyloidoses represent a group of disorders in which insoluble proteins deposit in tissues, resulting in damage to involved organs.^[1]^ Secondary (AA) amyloidosis is one of the most common forms of amyloid diseases.^[2]^ The amyloidogenic protein, AA type amyloid fibrils, is a proteolytic fragment of serum amyloid A protein (SAA), which is produced by the liver as an acute phase reactant. In AA amyloidosis, the deposition of AA type amyloid fibrils is responsible for the loss of function of the involved organs, including the kidneys, gastrointestinal tract, liver, heart, and the peripheral nerves.^[3]^

AA amyloidosis can occur during the course of many chronic inflammatory and infectious diseases.^[4]^ The etiology of AA amyloidosis differs in different geographies. Familial Mediterranean fever (FMF) is the most common cause of AA amyloidosis in Turkey,^[5]^ whereas rheumatoid arthritis (RA), juvenile idiopathic arthritis (JIA), ankylosing spondylitis (AS), and inflammatory bowel diseases are more common causes in Western countries.^[6]^ Infectious diseases, especially tuberculosis, are still the most common predisposing disease in developing countries, with a declining trend.^[7]^ There is still no definite treatment for amyloidosis. For many years, suppression of the inflammation by treating the underlying disease was the main strategy in AA amyloidosis. This provided a reduction in acute phase reactants, including SAA, resulting in the stabilization or even regression of amyloid deposition.^[6]^ Several agents have been used for this purpose. Colchicine, one of the most commonly used drugs for this purpose, has shown some benefit in both preventing and controlling AA amyloidosis in FMF patients.^[8]^ However, its beneficial effect in the other causes of AA amyloidosis is not clear.^[9]^ In addition, case reports and small case series involving patients with FMF and other rheumatic diseases have reported some success with various immunosuppressives and immunomodulators such as azathioprine,^[10]^ chlorambucil,^[11]^ methotrexate,^[12]^ interferon-alpha,^[13]^ and cyclophosphamide.^[14]^ In the last decade, biologic agents targeting proinflammatory cytokines such as tumor necrosis factor alpha (TNF-α), interleukin (IL)-1, and IL-6 have been tried in patients with AA amyloidosis.^[15–17]^ A more desirable approach for the treatment of amyloidosis would be targeting amyloid deposits. However, no such agents are currently available. Eprodisate inhibits the development of amyloid deposits by interfering with amyloidogenic proteins and glycosaminoglycans.^[18]^ However, it was recently announced that in the Phase III trial, eprodisate did not meet the primary endpoint in slowing renal function decline (http://www.bellushealth.com/English/investors-and-news/press-releases/press-release-details/2016/BELLUS-Health-announces-top-line-Phase-3-results-of-KIACTA-for-the-treatment-of-AA-amyloidosis/default.aspx). Another agent that targets amyloid deposits is R-1-[6-[R-2-carboxy-pyrrolidine-1-yl]-6-oxo-hexanoyl] pyrrolidine-2-carboxylic acid (CPHPC) that binds circulating serum amyloid-P (SAP) component and leads to a rapid clearance of SAP. An open label uncontrolled small study with CPHPC administration followed by infusion of a fully humanized monoclonal immunoglobulin G1 anti-SAP antibody, showed reduction of amyloid deposits in almost half of the patients.^[19]^ However, no data are yet available on the efficacy of this strategy in improving or preserving renal function in amyloidosis patients.

Anti-tumor necrosis factor alpha agents (anti-TNFs) show strong anti-inflammatory properties and have changed the disease course in several rheumatologic conditions such as RA and AS. Food and Drug Administration approved their use in patients with AS, RA, and inflammatory bowel diseases. Their off-label uses are also common in other rheumatologic disorders. The rationale for using anti-TNFs in AA amyloidosis depends on the fact that TNF-α is a potent inducer of SAA. Several case reports^[20–22]^ and a few of case series^[15,23–25]^ have reported clinical improvement or complete remission of signs of amyloidosis with anti-TNFs. However, these studies mostly had a small sample size with a relatively short follow-up. It is hard to attribute the beneficial course to the use of anti-TNFs without knowing the long-term outcome due to the slowly progressing natural course of AA amyloidosis. Moreover, a publication bias may be operative since most of these publications are case reports of few patients. The same concerns hold true for reports on other biologic agents such as IL-1 and IL-6 antagonists in AA amyloidosis.

The purpose of this study was to evaluate the long-term efficacy and safety of anti-TNFs, in patients with AA amyloidosis with different underlying inflammatory conditions, followed for up to 15 years in the rheumatology clinic of a university hospital.

## 2 Methods

We identified 37 AA amyloidosis patients who were started an anti-TNF for AA amyloidosis between March 2001 and June 2008 and followed until May 2016 unless deceased. We did not include 1 patient with AS and FMF who had a diagnosis of AA amyloidosis after a period of 7 years with adalimumab therapy. He is still on hemodialysis for 3 years.

We reviewed the charts of all patients in our clinic who had biopsy-proven AA amyloidosis for demographic features, primary diagnoses, disease and treatment duration, previous and concomitant medications, and adverse events. We evaluated them in the clinic for their final condition at the time of this study.

The endpoints were death, development of end-stage renal disease (ESRD) requiring renal replacement therapy (dialysis or renal transplantation) and change to another group of biologic agent due to worsening of amyloidosis indicated by an increase in proteinuria.

Patients who switched from one anti-TNF to another were scrutinized for the reasons of switch and response to the second or third anti-TNF agent. The anti-TNFs that were used in our clinic during this period were infliximab, etanercept, and adalimumab. The ethics committee of Cerrahpasa Medical Faculty approved this study.

## 3 Results

Among the 37 patients who had been treated with anti-TNFs for AA amyloidosis between March 2001 and June 2008, 25 were men and 12 were women. The mean age at disease onset and at the start of anti-TNFs, primary diagnoses, disease durations, and previous and concomitant drugs are given in Table 1. Angiotensin receptor inhibitors (angiotensin converting enzyme [ACE]) were given to 24 (65%) patients. Among the 13 patients who were not treated with ACE inhibitors, the reasons for this were proteinuria being lower than 1000 mg/d in 8 patients, serum creatinine level being higher than 3 mg/dL in 4 patients, and hypotension in 1 patient.

**Table 1:**
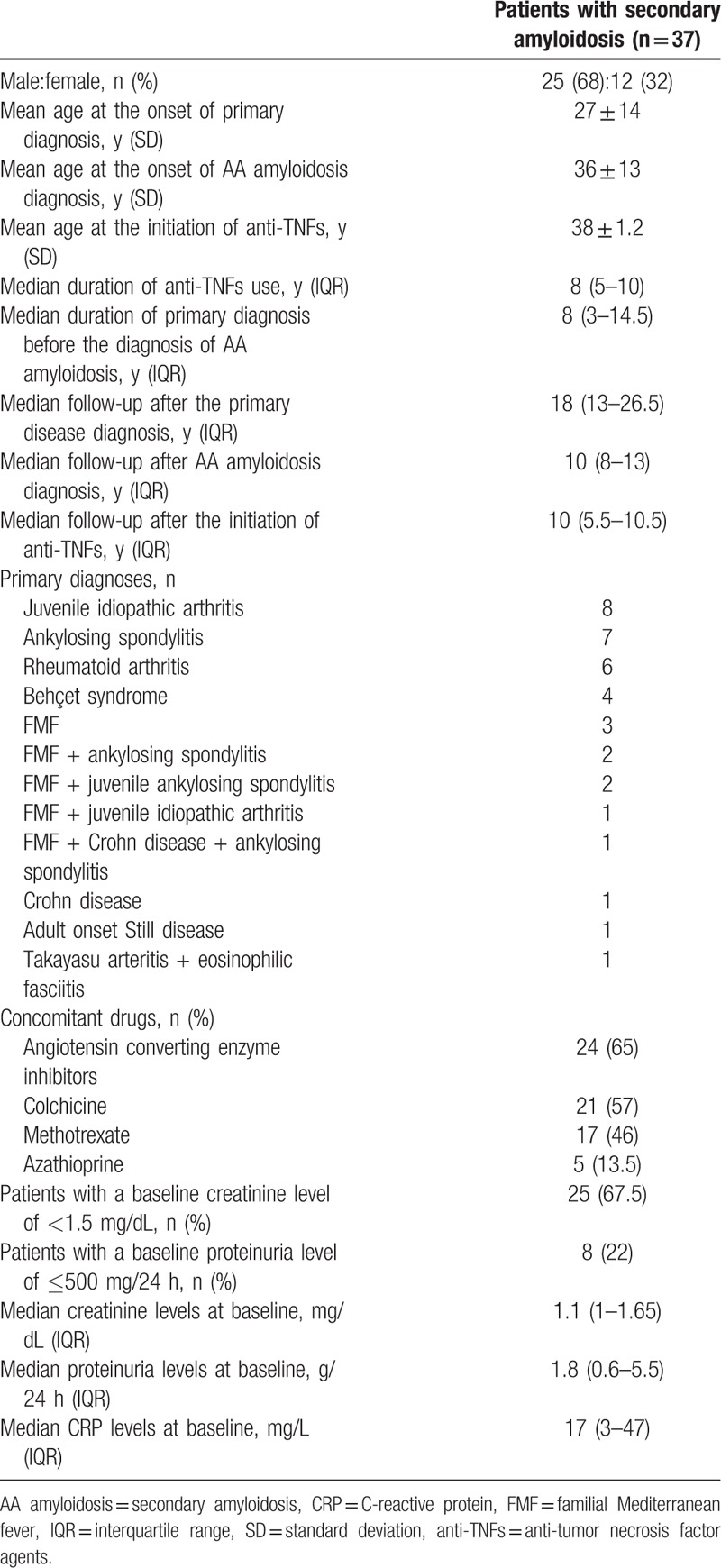
Demographic and clinical characteristics of 37 secondary amyloidosis patients.

Anti-TNFs were started immediately after the diagnosis of AA amyloidosis in 27 patients. The median time from the diagnosis of biopsy-proven AA amyloidosis to the beginning of treatment with anti-TNFs was 4 years (interquartile range [IQR]: 1.5–9.5) in the remaining 10 patients. The median duration of anti-TNFs use was 8 years (IQR: 5–10).

AA amyloidosis was confirmed with rectal biopsy in 21 patients, renal in 11 patients, lymph node in 1 patient, large bowel in 1 patient, stomach biopsy in 2 patients, and histopathological examination of the renal tissue resected for xanthogranulomatous pyelonephritis in 1 patient. AA amyloidosis was shown with Congo red staining in all patients. Immunohistochemistry was not available. The initial anti-TNF was infliximab in 27 patients and etanercept in 10 patients. Among the 27 patients receiving infliximab initially, 11 were switched to etanercept and 1 was switched to adalimumab. Of these 11 patients who were switched to etanercept, 1 patient then received infliximab again and 1 was switched to adalimumab. The reasons for switching from infliximab to another anti-TNF were ongoing underlying disease activity (n = 5), worsening of amyloidosis (n = 4), anaphylaxis (n = 4), aspergilloma (n = 1), and jugular and subclavian thrombosis (n = 1). Among the 10 patients who received etanercept initially, the second agent was infliximab in 2 patients and adalimumab in 3 patients. The reasons for switching were ongoing underlying disease activity (n = 4) and financial reason (n = 1).

### 3.1 Endpoints

**Figure 1:**
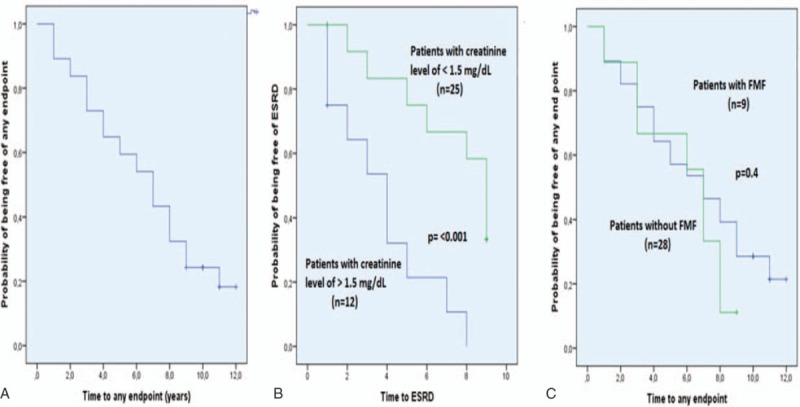
Kaplan–Meier graphs show the probability of being free of any endpoint (A), the probability of being free of end-stage renal disease in patients who had a baseline level of <1.5 and ≥1.5 mg/dL (B) and the probability of being free of any endpoint in patients with and without FMF (C). FMF = familial Mediterranean fever.

Among the 37 patients, 12 (32%) had died, 9 (24%) had ESRD, and 8 (22%) had started another group of biologic due to worsening of amyloidosis indicated by an increase in proteinuria at the end of a median follow-up of 10 years (IQR: 5.5–10.5) since the start of anti-TNFs and 10 years (IQR: 8–13) since the diagnosis of AA amyloidosis. The median time to any of the endpoints of death, development of ESRD or switch to another agent whichever is first was 5.5 years (IQR: 1.3–7.8) after initiation of an anti-TNF (Fig. 1A). The remaining 5 (14%) patients are continuing anti-TNFs for a median duration of 10 years (IQR: 10–10.5) and 3 (8%) are free of drugs as explained below.

#### 3.1.1 Death

Twelve (32%) patients had died after a median follow-up of 4 years (IQR: 2.5–5.5) after the initiation of anti-TNFs. Among the 12 patients who died, 2 had had a baseline creatinine level of ≥1.5 mg/dL and 8 had developed ESRD before death (Table 2).

**Table 2:**
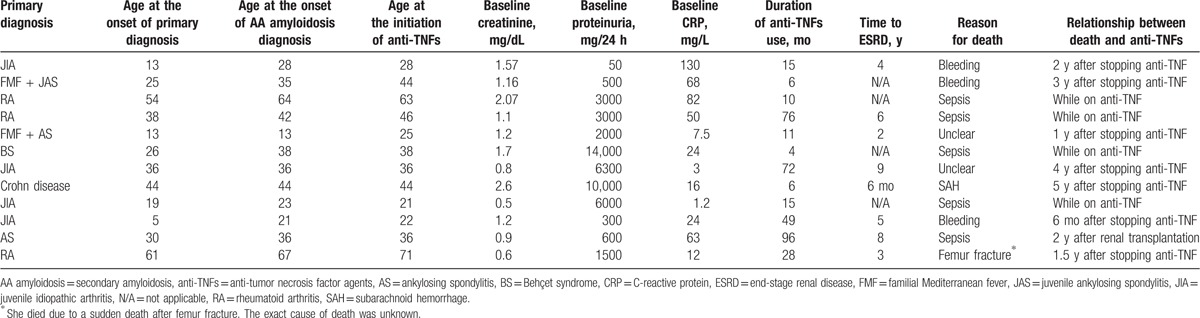
Demographic and clinical characteristics of 12 secondary amyloidosis patients who died.

The reasons for death were sepsis in 5 patients, bleeding in 3 patients, subarachnoid hemorrhage in 1 patient, unknown in 2 patients, and sudden death after femur fracture in 1 patient in whom the exact cause of death was not known. Among the 5 patients who died with sepsis, 4 were on an anti-TNF at the time of death. Those agents were infliximab in 2 patients, etanercept in 1 patient, and adalimumab in 1 patient. Of these, 2 patients with a baseline creatinine level ≥1.5 mg/dL died within 4 and 10 months after the initiation of anti-TNFs. Among the 5 patients who died due to sepsis, 2 had developed ESRD. One was under infliximab therapy for 8 years and renal function was stable for 7 years. When he developed ESRD, infliximab was stopped and peritoneal dialysis was started. This resulted in pseudomonal peritonitis and he was first switched to hemodialysis and later had renal transplantation. He died due to pneumonia 2 years after renal transplantation despite his renal function had been normal. The second patient had used infliximab for 4 years followed by adalimumab for a year and etanercept for a year and developed ESRD and died due to sepsis within 3 weeks.

None of the 3 patients who died with bleeding was using anti-TNFs at the time of bleeding. Two of them were undergoing hemodialysis and had massive bleeding from bladder 6 months and 3 years after stopping anti-TNFs. The third patient died with bleeding following fondaparinux use after hip prosthesis surgery, 2 years after she had stopped infliximab and had a stage 3 renal disease at the time of bleeding.

Among the remaining 4 patients who had died, 1 had used etanercept for 22 months and adalimumab for 6 months before the initiation of renal replacement therapy and was undergoing hemodialysis for a year at the time of death due to femur fracture. One patient who had a baseline creatinine level of 2.6 mg/dL before the initiation of infliximab developed ESRD after 6 months and was on hemodialysis for 5 years at the time of death due to subarachnoid hemorrhage. In the remaining 2 patients, the reason for death was not clear. In 1 of them, renal function had been stable under infliximab for 5 years followed by etanercept for a year and treatment was stopped due to a suspicion of malignancy, but this was not confirmed. The patient did not consent to restarting anti-TNF, developed ESRD, and died 4 years after stopping anti-TNF. The other developed ESRD 11 months after the initiation of infliximab and died a year later.

#### 3.1.2 End-stage renal disease

A total of 18 (49%) patients, including the 8 patients who later died due to other reasons explained above, had developed ESRD after a median follow-up 4.5 years (IQR: 2–8) after the initiation of anti-TNFs. One of these patients developed ESRD after switching to canakinumab due to increased proteinuria as explained below. Among the 25 patients who had a baseline creatinine level of <1.5 mg/dL, 8 developed ESRD and among the 12 patients who had a baseline creatinine level of ≥1.5 mg/dL, 10 developed ESRD. The estimated median time from anti-TNFs initiation to ESRD was 9 years (95% confidence interval [CI]: 7.9–10.1) among those with a baseline creatinine level of <1.5 mg/dL compared with 4 years (95% CI: 2.2–5.8) among those with a baseline creatinine level of ≥1.5 mg/dL (*P* < .001; log-rank) (Fig. 1B).

Among the 9 patients who are still alive, 8 patients started to undergo hemodialysis and 2 of them received renal transplantation 2 and 8 years after the initiation of hemodialysis. The last patient underwent preemptive renal transplantation 5.5 years after starting infliximab. Anti-TNF therapy was stopped in all but 1 patient when the renal replacement therapy was started. Only 1 patient with AS who is undergoing dialysis is still continuing etanercept therapy. Among the 3 patients who received renal transplantation, 1 with Behçet syndrome (BS) is still using tocilizumab for 2 years that was started after transplantation, 1 with AS is using adalimumab for 4 years that was started after transplantation and the third patient with JIA is not receiving any biologic agents for a year. Renal function is stable in all 3 patients.

#### 3.1.3 Switch to another biologic agent

A total of 8 patients were switched to IL-1 antagonists (n = 5), IL-6 antagonists (n = 2), and rituximab (n = 1) due to increase in proteinuria after a median follow-up of 7.5 years (IQR: 7–8.5) after the initiation of anti-TNFs. One of these patients developed adenocarcinoma of the lung and later ESRD. He had used infliximab for 14 months, etanercept for 48 months, and canakinumab for 3 months before developing lung carcinoma. Canakinumab was stopped, chemotherapy and radiotherapy were started, and ESRD developed 4 months later. One patient with juvenile onset AS and FMF who was started anakinra after anti-TNF was later switched to tocilizumab due to active joint involvement. Six patients are currently stable with canakinumab (n = 3) and tocilizumab (n = 3). In addition, 1 patient was given rituximab for severe RA after a period of 6.5 years with etanercept therapy, and her proteinuria and renal functions are also stable with this treatment for 4 years.

### 3.2 Continuing anti-TNFs

The remaining 5 (14%) patients are still doing well with anti-TNFs at the end of a mean follow-up of 9.5 ± 1 years. The primary diagnoses were AS in 3 patients, adult onset Still disease in 1 patient, and eosinophilic fasciitis and Takayasu arteritis in 1 patient. All patients had a baseline creatinine level of <1 mg/dL at the time of initiation of etanercept (n = 3) and infliximab (n = 2). Proteinuria was present in 4 patients, within the nephrotic range in 2 patients. Proteinuria disappeared in all of these patients.

### 3.3 Patients who are off-treatment

Three (8%) patients were off-treatment at the time of survey. The diagnoses of these 3 patients were JIA, BS, and FMF together with AS. Anti-TNF therapy was stopped after 6, 6.5, and 9 years due to stable renal disease without proteinuria. They are still off-treatment for 1.5, 3, and 4 years. These were relatively mild patients at baseline. All of them had a baseline creatinine level of <1 mg/dL at the time of initiation of anti-TNFs. Two of them had a non-nephrotic range proteinuria at baseline that resolved with therapy. The last patient with BS neither experienced a proteinuria nor a worsening of creatinine during a follow-up of 11 years.

### 3.4 Outcomes according to primary diagnosis

The treatment duration among patients without FMF (n = 28) and those with FMF (n = 9, 3 FMF only, and 6 FMF concomitant with AS or JIA) was not significantly different (43 ± 30 months vs. 66 ± 43 months, *P* = .15). When we compared the treatment outcome among patients with and without FMF, there was no difference regarding the endpoints of death, ESRD, or switch to another drug due to worsening of amyloidosis (7 years [95% CI: 5.6–8.3] vs. 7 years [95% CI: 3.9–10.1], *P* = .4; log-rank) (Fig. 1C).

### 3.5 Adverse events

Overall 20 adverse events that were necessitating cessation of anti-TNFs, or requiring hospitalization or death occurred in 15 patients. Infections (n = 10) were the most common serious adverse event, followed by anaphylaxis with infliximab in 4, vein thrombosis in 3, arterial thrombosis in 1, vasculitis in 1, and adenocarcinoma of the lung in 1.

The serious infections had occurred while on infliximab in 8 patients, on adalimumab and on etanercept in 1 patient each. The serious infections were sepsis in 8 patients; 4 of them had died with sepsis while on infliximab in 2 patients, on adalimumab and on etanercept in 1 patient each, as mentioned above. One further patient died with sepsis 4 years after stopping infliximab who had undergone renal transplantation 3 years before. One of them had a previous lumbar abscess and pneumonia, 2 and 4 years after the initiation of infliximab, respectively. Among the remaining 2 patients who had sepsis, 1 had *Escherichia coli* pyelonephritis and the other had tuberculosis, resulting in ESRD in both. In addition, a gluteal abscess and aspergilloma requiring hospitalization occurred in 1 patient each.

A total of 4 patients experienced thrombotic events under infliximab therapy. The involved vessels were retinal vein (n = 1), inferior vena cava and renal vein (n = 1), internal jugular and subclavian vein (n = 1), and popliteal artery (n = 1). The primary diagnoses of these 4 patients were JIA, BS, AS, and RA, respectively.

There was no difference regarding the amount of proteinuria in patients who experienced or who did not experience adverse events while being on IFX (2450 mg/d [IQR: 775–4100] vs. 1600 mg/d [550–4050]; *P* = .54). Seven of the 12 patients who experienced adverse events did not have nephrotic range proteinuria.

Four patients experienced anaphylaxis during infliximab infusions. One patient who had FMF, Crohn disease, and AS developed vasculitic skin lesions and neuropathy while on infliximab treatment. Cyclophosphamide was started and later switched to tocilizumab when vasculitic lesions disappeared. One patient who was switched to canakinumab developed adenocarcinoma of the lung as explained above.

## 4 Discussion

The lack of currently available agents that directly target amyloid deposits mandates the use of agents that strongly suppress the inflammation caused by the primary disease. Biologic agents including anti-TNFs, IL-1, and IL-6 blockers are the main therapeutic options used for this purpose. A retrospective study that indirectly compared tocilizumab to anti-TNFs, with a median treatment duration of 2 years suggested a more favorable outcome with tocilizumab.^[26]^ Although IL-6 blockage seems to have the advantage of significantly reducing circulating SAA levels, its long-term impact on renal function is not known. Moreover, switching between these agents is frequently necessary in inflammatory conditions due to adverse events and primary or secondary inefficacy.^[27]^ Thus, information on the long-term efficacy and safety of these agents would help to develop management strategies in patients with secondary amyloidosis.

This observational study of AA amyloidosis patients with different underlying diseases followed for a median duration of 10 years after starting anti-TNFs showed that 32% of the patients had died, 24% had ESRD, and 22% were switched to another group of biologic agent due to increase in proteinuria. Only 14% of the patients are still using anti-TNFs for a median duration of 10 years. Several adverse events such as severe infections (n = 10), anaphylaxis (n = 4), and thrombosis (n = 4) that can be attributed to anti-TNFs and/or amyloidosis itself have been observed.

Determining the natural course of AA amyloidosis has been a challenging issue due to several reasons. First, it is thought that a long time is required for clinical manifestations of amyloidosis to become overt, after amyloid deposition has started. However, the length of this time has not been well established and may vary largely between patients.^[28,29]^ Second, most of the papers that include small numbers of patients with different underlying diseases have reported relatively short follow-up durations which makes it difficult to elaborate on the disease course. Third, lead time bias may complicate the understanding of the benefits of early diagnosis and treatment on the prognosis of AA amyloidosis.

The most comprehensive study on the course of AA amyloidosis had been reported by Lachmann and colleagues among 374 patients followed between the years 1990 and 2005.^[30]^ This study showed that 44% of the patients died, and 33% developed ESRD during a median follow-up of 86 months after diagnosis. Detailed information on treatment is not provided in this study, but we understand from their demographic table that <60% of their patients were treated with a biologic including anti-TNFs. The survival rates were even worse in series coming from nephrology in which patients were referred with high creatinine levels.^[31–33]^ Compared to the Lachmann study which reported 56% survival over a median follow-up of 86 months after the diagnosis of AA amyloidosis, our survival rate of 68% over a median follow-up of 126 months may indicate an improvement in the prognosis of AA amyloidosis patients with anti-TNFs. This finding may be confounded by the fact that our patients were younger when diagnosed with AA amyloidosis compared with Lachmann cohort (35 years vs. 50 years). Another study by Kuroda et al reported a significantly higher survival rate among patients with AA amyloidosis due to RA who received biologics compared with those who did not.^[34]^ Moreover, etanercept was found to be more effective than cyclophosphamide in improving survival of RA patients.^[35]^

Apart from case reports, there were 4 case series reporting on the use of anti-TNFs for patients with AA amyloidosis. The first retrospective case series by Gottenberg and colleagues, published in 2003, suggested that anti-TNFs were well tolerated, safe and a potential treatment option for AA amyloidosis.^[24]^ However, making a decision on the safety and tolerability of anti-TNFs was difficult since the mean follow-up of those 15 patients was 10 months. Similar favorable outcomes of 14 subjects in each study were observed by Nakamura and colleagues^[36]^ and by Kuroda and colleagues,^[23]^ but again with a relatively short follow-up of 1.5 to 2 years. Fernández-Nebro and colleagues conducted a multicenter prospective study involving 36 AA amyloidosis patients and showed that 22% of the patients had died, 17% had kidney progression, and 52% of the subjects remained on anti-TNFs, at the end of a median follow-up of almost 3 years.^[15]^

It is unclear which rheumatic disease complicated with AA amyloidosis responds well to anti-TNFs. The underlying diseases of AA amyloidosis patients who were involved in the previously reported anti-TNF series were almost always RA or AS. In our survey coming from a country where FMF is prevalent and is the most common underlying cause of amyloidosis,^[5]^ we had 3 patients with FMF and 6 patients with rheumatic diseases concomitant with FMF, different from the other series. A favorable effect on amyloidosis can be expected in RA and AS patients since TNF-α is a strong inducer of inflammation in these diseases,^[37]^ SAA was suggested to be a good marker of RA disease activity, and SAA levels were decreased with anti-TNF treatment in RA patients.^[38]^ However, the experience with anti-TNFs in patients with FMF was extremely limited.^[39]^ We observed a similar outcome with anti-TNFs in our AA amyloidosis patients with and without FMF.

Despite having many benefits, anti-TNFs have well-known adverse effects. Among them, infections are the most common adverse event that are also the leading cause of death in AA amyloidosis patients.^[40]^ Moreover, Fernández-Nebro and colleagues showed a 3-fold increase of infections in AA amyloidosis patients receiving anti-TNFs, compared to propensity score-matched population free of amyloidosis.^[15]^ As expected, infections were the most frequent adverse event among our patients that led to death in 4/10 and to ESRD in 2/10. Among the infections, reactivation of latent tuberculosis is a major concern with anti-TNFs, as we also observed in 1 of our patients in this series. However, the continuation of biologic therapy may be considered in patients undergoing renal transplantation to prevent disease recurrence in the transplanted kidney and to control the underlying inflammatory disease.^[41]^

The main limitations of our study were its retrospective design and the lack of follow-up of SAA levels under anti-TNF treatment, which would provide more information on the efficacy of these agents on amyloidogenesis. In addition, only 30% of the patients were diagnosed by renal biopsy that can be considered gold standard for diagnosing renal amyloidosis. Our routine practice is to initially perform rectal biopsy in patients with a suspicion of amyloidosis. This is due to the fact that rectal biopsy is a relatively safe procedure compared with renal biopsy.

In conclusion, our long-term observational study together with previous studies of AA amyloidosis patients treated with and without biologic agents shows that the prognosis of AA amyloidosis may have improved in the biologic era. Starting these agents early, while serum creatinine level was <1.5 mg/dL in our series, may delay the progression to ESRD. However, caution is needed regarding serious adverse events, especially infections. Whether an anti-TNF, IL-1 blocker, or IL-6 blocker should be preferred in an individual patient depending on the primary diagnosis or other patient characteristics remain to be studied.
